# Who Benefits from Home Treatment? Predictors of Treatment Outcome in an Acute Psychiatric Setting: an Observational Study

**DOI:** 10.1007/s11126-025-10131-z

**Published:** 2025-04-02

**Authors:** Felix Baumann, Vera Bergamaschi, Ingeborg Warnke, Salvatore Corbisiero, Fabian Ludwig, Andreas Riedel, Kerstin Gabriel Felleiter, Hansjörg Znoj, Stefanie J. Schmidt

**Affiliations:** 1 Lucerne Psychiatry, Ambulatorium B, Löwengraben 20, 6004 Lucerne, Switzerland; 2 Institute of Psychology, University of Berne, Bern, Switzerland

**Keywords:** Mental health services, Acute psychiatry, Home treatment, Predictors

## Abstract

Systematic reviews have confirmed that home treatment (HT) is an appropriate alternative to conventional inpatient treatment. So far, research on predictors for treatment outcomes of HT has been rather inconsistent, and potential predictors have not been systematically investigated yet. This exploratory study has a prospective naturalistic design with repeated measurements of symptoms, well-being, and self-efficacy at baseline, at post-assessment (discharge from HT) and at follow-up. Repeated measures analysis of variance (ANOVA) was carried out to measure changes between assessment points. Changes in emotion regulation were analysed between baseline and post-assessment using t-tests. Potential predictor variables were assessed at baseline. Linear regression models were estimated with post scores of symptoms, well-being, self-efficacy and emotion regulation as dependent variables and potential predictors as independent variables. 58 patients participated in the study. Significant differences between baseline and post-assessment were found for psychiatric symptoms, well-being, self-efficacy and emotion regulation. No significant changes were found at follow-up. Of the investigated potential predictors, three significantly predicted outcome of HT: Patients with former treatments in a psychiatric institution had significantly higher post-assessment in symptoms (*β* = .26, *p* = *.04*) and lower well-being (*β* = -.28, *p* = *.02*) compared to patients without former treatment in a psychiatric institution. Furthermore, hope for change and symptoms of anxiety were found to be predictors of outcome. General improvement in symptoms and well-being indicate that HT was effective. Previous psychiatric history, hope of improvement and anxiety were identified as predictors of treatment outcome.

## Introduction

Home treatment (HT) offers an intensive acute psychiatric treatment in the patients’ domestic environment. It is delivered to patients in an acute psychiatric crisis, who would otherwise be treated in a psychiatric hospital. The basis of HT is a mobile and interdisciplinary team of psychiatrists, psychologists and nurses. Patients are visited at home daily for a limited treatment period. An emergency phone is provided to ensure 24-h availability [16]. Systematic reviews have confirmed the benefits of HT as an appropriate alternative to conventional inpatient treatment. For example, studies found that the implementation of HT reduced the number of inpatient treatments during and after an acute psychiatric crisis [14, 24]. The reduced need of inpatient treatment after the implementation of HT was also supported in a recent randomized controlled trial. Over 700 adults in need of acute psychiatric treatment were either admitted to a conventional in-patient setting or an HT service. HT patients needed 30% fewer hospital days within two years after the initial crisis compared to the patients in in-patient care [31]. Notably, the definition of relevant outcomes within the context of HT requires further attention. Besides the mentioned reduction of hospital days, fewer studies have investigated clinical variables during HT as indicators for treatment success. In a recent study in Switzerland, a reduction of symptoms and an improvement in patients’ level of functioning during HT were found [26]. The role of self-efficacy defined as the expectation of an individual to be able to cope with or overcome a difficult situation or obstacle on their own [1] within HT was investigated in a Dutch study [2]. Therein, an increase in self-efficacy during treatment was associated with an improvement in symptoms.

Another clinical variable that may be relevant in HT is emotion regulation. A systematic review analysed the role of emotion regulation defined as a collection of strategies that an individual uses to increase, maintain or decrease the current emotional experience as a transdiagnostic treatment element across different mental illnesses [30]. The review investigated whether changes in emotion regulation occurred during the treatment of various mental disorders and whether these changes were associated with a positive therapeutic outcome. There were significant reductions in maladaptive emotion regulation strategies for most patients during the treatment, regardless of the therapy method used or the disorder treated.

Against this background in the present study, changes in symptoms and well-being were defined as primary outcomes for treatment success. Well-being was chosen as outcome variable as health is often seen as more than the absence of symptoms, as discussed by Bech et al. [5]. To examine a broader range of outcomes, changes in self-efficacy (see [2]) and emotion regulation were also investigated. Based on previous findings, it was assumed that there were significant improvements in symptoms, well-being, self-efficacy and emotion regulation.

While the effectiveness of HT is well documented, less is known about whether certain patient characteristics are associated with a better outcome of HT (i.e., differential indication). This knowledge would help to know who may benefit most from HT. Only a few studies have performed secondary analyses to identify potential predictors of HT outcomes (e.g., [17, 22]). These findings were recently summarized in a systematic review [6]. Therein, the following variables predicted better outcomes in terms of changes in clinical variables during HT: lower age, living in less socially deprived areas, being employed, being less lonely, family is involved in the treatment, lesser or no previous hospitalisations, level of functioning (mixed effect), lower severity of symptoms, no substance abuse and no / low risk for suicidality. However, previous results on predictors of treatment success are inconsistent and were often only a by-product of general efficacy studies. Therefore, the aim of the present study was to address the question of predictors as primary research question. To answer the question about potential predictors for treatment outcome in HT, the following variables were assessed: sociodemographic variables (age, sex, civil status, living situation, work status, highest degree), psychiatric history, well-being, symptoms, self-efficacy, emotion regulation (adaptive and maladaptive strategies) and therapy expectation (hope of improvement, fear of change, suitability). According to previous findings [4] it was hypothesized that younger age, female gender, symptoms of anxiety, fewer number of previous hospitalizations and living with others serve as predictors for the treatment success To the best of our knowledge, therapy expectation has not yet been investigated as a predictor in the field of HT but has found to be a predictor of treatment outcomes in other therapeutic approaches [13].

## Method

### Design

An exploratory, prospective, naturalistic design was used. All data were collected via questionnaires during the regular treatment of an existing HT service. Baseline assessment took place before entering HT. At baseline, the potential predictor variables were assessed. To assess treatment outcome, the selected clinical variables were assessed at baseline, at discharge (i.e., post) and at follow-up (i.e., three months after discharge).

### Setting

The study took place in a home treatment service offered by Lucerne Psychiatry in Switzerland. The treatment does not target patients with a specific disorder but is open to acute psychiatric patients from the entire psychiatric diagnosis spectrum. Patients were visited daily in their domestic environment. The interdisciplinary treatment team consisted of psychiatrists, psychologists and psychiatric nurses. To ensure treatment consistency, the team met daily to report and discuss the course of treatment. An emergency phone ensured 24 h availability of the team. In case of a psychiatric emergency, a bed was available in the nearby psychiatric clinic. If the patients required the bed for more than 7 days, they were transferred to inpatient treatment. Patients got a sickness certification during treatment. The treatment was limited to the acute phase of the symptoms.

### Sample

Patients in need of an acute psychiatric treatment were referred to HT by clinical practitioners and institutions like psychiatric or somatic hospitals. Patients had to be able to distance themselves from acute suicidal tendencies and endangerment of others as a prerequisite for treatment. Minimal age for treatment was 18 years. The only other inclusion criteria for the study were voluntary participation (including informed consent) and German language proficiency. During the recruitment period from June 2021 – June 2023, 58 patients were included in the final sample.

### Procedures and Measures

The study protocol and procedures were approved by the Swiss ethics committee Nordwest- und Zentralschweiz (EKZN: 2020–02736) and published [4]. After referral for treatment, patients were contacted and informed about the study. Participation was not compensated. Written and verbal consent was obtained. The following variables were assessed at baseline as potential predictor variables: Sociodemographic data (age, sex, civil status, living situation (alone/with others), work status (working/not working), highest degree of education, previous psychiatric treatments (previous psychotherapy yes/no and previous treatment by a psychiatric institution yes/no). Therapy expectation was assessed with the”Messung der Therapieerwartung und Therapieevaluation von Patienten” (PATHEV, [27]). The PATHEV consists of three subscales: hope of improvement, fear of change and perceived suitability of treatment. The German version of the Brief Symptom Checklist (BSCL, [12]), which consists of subscales for hostility, anxiety, depression, paranoid ideation and phobic anxiety, psychoticism, somatization, interpersonal sensitivity, and compulsivity, the German version of the World Health Organization-Five Well-Being Index (WHO-5, [5]) served to assess well-being, the “Scale for General Self-Efficacy Expectations” (SWE, [28]) self-efficacy and the “Questionnaire for Emotion Regulation in Difficult Life Situations” (EMOREG, [34]) emotion regulation. The EMOREG consists of subscales for adaptive and maladaptive emotion-regulation. Therein, adaptive emotion-regulation is defined as self-control, in which the regulation of strong emotions occurs automatically, and expression, the regulation of mood through verbal expression. Maladaptive emotion-regulation is defined as avoidance in the form of distraction, constant preoccupation or breaking off social relationships as well as distortion, i.e., the attempt to regulate one's state by drastic means such as observing oneself or others or misunderstanding or feeling misunderstood. The sum score was used for the analysis of the questionnaires and their subscales.

The outcome HT was assessed with baseline, post- and follow-up measures of BSCL, WHO-5, SWE and baseline and post-measures of EMOREG. All questionnaires could be completed either online or in a paper-and-pencil version.

### Data Analysis

Repeated measures analysis of variance (ANOVA) was carried out to measure changes between baseline, post-assessment and follow-up of symptoms, well-being and self-efficacy. For the ANOVA, a power analysis indicated that a sample size of 43 people was sufficient to demonstrate a mean effect of f = 0.25. Mauchly’s Tests were used to test for the assumption of sphericity. A post-hoc analysis was used to check whether there are significant differences between the individual measurement times. Changes in emotion regulation between baseline and post-assessment were calculated using a t-test for paired values.

Large units of missing data were replaced using multiple imputation, if data were missing at random. For the imputation, 50 data sets with estimates for the values of the missing data were created with SPSS (2023, version 29) and pooled for further calculations. If missing measurements were not random, only complete measurements were used and differences between participants with missing measurements and participants with complete measurements were examined.

To test for the effect of potential predictors on treatment outcome, post-assessment of symptoms, well-being, self-efficacy and emotion-regulation as dependent variables and potential predictors as independent variables were entered in linear regression models. The respective baseline variables were added as covariates. The same regression models were used for the follow-up values of these outcome variables (except for emotion-regulation). Additional regression models were carried out using the subscales of the BSCL as independent variables to predict post values of well-being, self-efficacy and emotion regulation. For all the regression models, baseline values of the respective outcome variables were entered as covariates. Kolmogorov–Smirnov/ Shapiro–Wilk tests were carried out to test for normal distribution of residuals for all regression models and Durbin-Watson for autocorrelation. Collinearity diagnostics for these regression models were carried out to test for multicollinearity [11]. Data were analysed by IBM SPSS Statistics (2023, Version 29).

## Results

### Sample Characteristics

During the recruitment period from June 2021 – June 2023, a total of 557 patients were admitted to the HT service under investigation. 58 patients could not be included due to insufficient German language proficiency. 22 patients were excluded because they were admitted for the second time during the study period and had already been asked to participate before. Five patients could not be recruited due to an emergency admission to HT. 156 patients could not be included because they could not be reached by phone before starting treatment or they canceled treatment before entry. The remaining 316 patients were asked to participate in the study. 226 patients declined participation. 90 patients signed the informed consent and were included in the study. Due to missing baseline or post-assessment or incomplete measurements, 32 participants could not be included in the final analysis. In one case, missing of baseline assessment of BSCL and in a second case missings of BSCL and WHO-5 could be replaced with data from assessment after one week of treatment. These additional assessments were part of part of the research project but generally not used in this study [4]. The final sample included 58 patients. Sociodemographic data, mean days from referral until beginning of HT, mean treatment duration and diagnostic characteristics of the included patient sample are presented in Table 1.

**Table 1:** Characteristics of the patient sample (*N* = 58)

Sociodemographic variables	Mean (*SD*)/ frequency (%)/ number
Age	40.81 (15.99)
Sex	Male: 19 (32.8%) Female: 39 (67.2%)
Civil status	Single: 26 (44.8%) Married: 19 (32.8%) Divorced/separated: 13 (22.4%)
Living situation	Alone: 16 (27.6%) With others: 42 (72.4%)
Working status	Yes: 30 (51.7%) No: 28 (48.3%)
Highest degree	Completion of regular schooling: 7 (12.1%) Apprenticeship: 30 (51.7%) Higher degrees: 21 (36.2%)
Psychiatric history	
First contact with psychiatry	Yes: 8 (13.8%) No: 50 (86.2%)
Former psychotherapy	Yes: 39 (67.2%) No: 19 (32.8%)
Former treatment by a psychiatric institution	Yes: 38 (65.5%) No: 20 (34.5%)
Mean days from referral until beginning of HT	15 (*SD* = *52.9*)
Mean days duration of HT	45 (*SD* = *22.9*)
Symptoms, brief symptom checklist subscales	
Hostility	1.18 *(0.76)*
Anxiety	1.71 *(0.96)*
Depression	1.95 *(0.92)*
Paranoid ideation	1.18 (0.82)
Phobic anxiety	1.60 *(1.20)*
Psychoticism	1.41 *(0.84)*
Somatization	1.22 *(0.82)*
Obsessive compulsive	2.16 *(0.83)*
Interpersonal sensitivity	1.69 *(0.89)*

Paired values

*M* Mean; *SD* Standard deviation

Of the 32 participants that were excluded due to missing measurements, 18 participants were excluded due to missing post-assessments. The group with missing post-assessments was the largest group among the participants with missing values. For the remaining 14 patents with missing values, the reasons were more heterogeneous, ranging from completely missing measurements to measurements with missing questionnaires or missing individual values. Baseline score of WHO-5 and BSCL as well as sociodemographic variables of the excluded 18 participants with missing post-assessments were compared with the final sample. Compared to the final sample, the 18 excluded participants showed no difference regarding sociodemographic variables. However, the excluded participants showed significantly higher symptom-scores at the beginning of treatment (m = 1.70, SD = 0.76) compared to the final sample (m = 1.59, SD = 0.69, *p* < *0.01)*. Therefore, missing data of post-assessments were not estimated using multiple imputation. In 28 of 58 cases included, the follow-up assessment was missing. Analysis of missing data of WHO-5, BSCL and SWE at follow-up indicated missing completely at random (Chi-square = 0.88, df = 3, *p.* = *0.83*). Missing follow-up data were therefore replaced using missing data imputation.

### Changes of Clinical Variables During HT

Mauchly’s tests for sphericity indicated no violation for the assumption of sphericity. The ANOVA with repeated measures showed significant differences between baseline, post- and follow-up in overall symptom levels, well-being and self-efficacy with large effects-sizes (see Table 2). However, post hoc analysis revealed no significant differences between post-assessment and follow-up.

**Table 2:** Changes in clinical variables

	Mean* (SD)*			Sum of squares	*df*	M	*F*	*p*	Partial η^2^
	Baseline	Post	Follow-up						
Well-being	1.25 *(1.03)*	2.23 *(1.16)*	2.20 *(0.81)*	36.03	2	18.02	30.67	< 0.01	0.35
Symptoms	1.59 *(0.69)*	1.04 *(0.67*)	1.07 *(0.42)*	11.17	2	5.59	30.64	< 0.01	0.35
Self-efficacy	2.28 *(0.63)*	2.57 *(0.60)*	2.44 *(0.44)*	2.44	2	1.22	9.14	0.01	0.14

ANOVA with repeated measures for baseline, post−− and follow−up

*SD* Standard deviation follow−up; *df* degrees of freedom; *M* Mean squares; *F* f−statistic; *p* probability value; *partial η2* effect size

There were significant improvements in the two adaptive subscales of the EMOREG self-control at baseline (M = 3.04, *SD* = 1.06) and post-assessment (M = 3.40, *SD* = 1.00), (t = 3.19, *p* < 0.01), and expression at baseline (M = 3.08, *SD* = 1.1) and post-assessment (M = 3.38, *SD* = 1.22), (t = 1.88,* p* = 0.03). For the maladaptive subscale of the EMOREG, there was a significant reduction between baseline (M = 3.79, *SD* = 0.928) and post-assessment (M = 3.29, *SD* = 0.96), (t = −4.06, *p* < 0.01). Effect sizes were medium (> 0.5) for the self-control and the maladaptive subscale of the EMOREG and small (> 0.2) for the expression subscale of the EMOREG.

### Analysis of Predictor Variables

Kolmogorov–Smirnov and Shapiro–Wilk tests indicated normal distribution of residuals for all regression models (*p* > 0.05). Durbin-Watson test indicated no autocorrelation. Collinearity diagnostics for these regression models indicated no multicollinearity [11]. As there was no significant difference between follow-up and post-assessment, predictor analysis was only carried out for the post-assessment.

Symptoms and well-being (see Table 3): Psychiatric history significantly predicted post-assessment of well-being and symptom severity. Patients with former treatments by a psychiatric institution had significantly higher post-scores in symptoms (*β* = 0.26, T = 2.17, *p* = *0.04*) and lower post scores in well-being (*β* = −0.28 T = −2.42, *p* = *0.02*) compared to patients without a history of former treatments by a psychiatric institution. Effect sizes were small for the prediction of BSCL (*β* = *0.26*) and for the prediction of WHO-5 (*β* = *0.28*). None of the other predictors examined were associated with symptoms and well-being. However, significant results were found in relation to the included covariates. Higher well-being at baseline was significantly associated with higher well-being at post assessment (*β* = 0.72, T = 4.87, *p* < *0.01*) and higher symptom severity at baseline were significantly associated with higher symptom severity at post-assessment (*β* = 0.48, T = 2.62, *p* = *0.01*).

**Table 3:** Analysis of predictors of well-being and symptoms

		Well-being					Symptoms				
		*B*	*SE*	*β*	*T*	*P*	*B*	*SE*	*β*	*T*	*P*
Sociodemographic variables	Age	0.06	0.01	0.08	0.57	0.57	−0.00	0.01	−0.05	−0.36	0.72
	Sex	0.56	0.29	0.23	1.94	0.06	−0.34	0.18	−0.24	−1.90	0.06
	Civil status *single/married/divorced/separated*	−0.16	0.32	−0.07	−0.52	0.61	−0.24	0.20	−0.17	−1.17	0.24
	living situation *alone/ with others*	0.28	0.33	0.11	0.87	0.39	0.17	0.20	0.12	0.88	0.39
	work status *working/ not working*	−0.01	0.30	−0.01	−0.04	0.97	−0.00	0.18	−0.002	−0.01	0.10
	highest degree	−0.28	0.20	−0.16	−1.41	0.17	−0.02	0.12	−0.02	−0.16	0.87
Psychiatric history	former psychotherapy *yes/no*	−0.04	0.29	−0.02	−0.13	0.90	0.03	0.18	0.02	0.19	0.85
	former treatment by a psychiatric institution *yes/no*	−0.67	0.28	−0.28	−2.42	0.0*2*	0.36	0.17	0.26	2.17	*0.04*
Well-being	WHO	0.81	0.17	0.72	4.87	0.00	−	−	−	−	−
Symptoms	BSCL	−	−	−	−	−	0.46	0.18	0.48	2.62	0.01
Self-efficacy	SWE	−0.65	0.38	−0.35	−1.72	0.09	0.17	0.24	0.16	0.73	0.47
Emotion regulation	EMOREG *self-control (adaptive)*	0.06	0.25	0.05	0.24	0.81	−0.07	0.13	−0.12	−0.52	0.61
	*avoid/distort (maladaptive)*	−0.16	0.18	−0.13	−0.92	0.36	0.05	0.13	0.08	0.43	0.67
Therapy expectation	PATHEV *hope of improvement*	0.13	0.30	0.05	0.45	0.66	−0.28	0.18	−0.18	−1.53	0.14
	*fear of change*	−0.28	0.19	−0.20	−1.49	0.14	0.03	0.11	0.04	0.26	0.80
	*suitability*	−0.16	0.31	−0.06	−0.51	0.61	−0.09	0.19	−0.05	−0.46	0.65

Linear Regression Model

*B* Non-standardized coefficient; *SE* Standard error; *β* Standardization factor, *T* t-value, *P* Probability value

Self-efficacy: Post-assessment of self-efficacy was significantly predicted by the PATHEV subscale hope of improvement (*β* = 0.39, t = 3.29, *p* < *0.01*). Patients with higher hope for improvement before HT had significantly higher self-efficacy scores at the time of discharge of HT compared to patients with lower hope for improvement.

Emotion regulation: Adaptive emotion-regulation (self-control) at post-assessment was significantly predicted by the PATHEV subscale hope for improvement (*β* = 0.31, T = 2.80, *p* = *0.01*). The covariate adaptive emotion-regulation was significantly associated with adaptive emotion-regulation at post-assessment (*β* = 0.64, T = 2.99, *p* < *0.01*). Maladaptive emotion-regulation was significantly predicted by a history of former treatment by a psychiatric institution (*β* = 0.26, T = 2.13, *p* = *0.04*).

Subscales of symptoms: In the additional regression models, the BSCL subscale anxiety (*β* = 0.42, T = 2.15, *p* = *0.04)* at the beginning of HT significantly predicted adaptive emotion regulation (self-control) after HT. Patients with higher anxiety at baseline showed higher adaptive emotion regulation at post-assessment, compared to patients with lower levels of anxiety. The included covariate of adaptive emotion regulation at baseline was significantly associated with adaptive emotion regulation at post-assessment (*β* = 0.42, T = 3.20, *p* < *0.01).* No other BSCL subscale significantly predicted post-assessment of well-being, self-efficacy, adaptive emotion regulation (expression) or maladaptive emotion regulation (see Appendix for the additional regression analyses).

## Discussion

This study is one of the first to address the question of predictors for the success of HT as a primary research question. Overall, HT was associated with a significant reduction in symptoms, increase in well-being, increased adaptive emotion-regulation, decreased maladaptive emotion-regulation and increased self-efficacy. These results are in line with previous studies focusing on changes in clinical variables during HT that showed a significant reduction in symptom severity [31] in particular in depressive and psychotic symptoms. The results regarding self-efficacy could be explained by a previous study discussing that HT strengthens patients’ confidence in their ability to take control of their situation and environment, thereby increasing their self-efficacy [2]. Results regarding improvements in emotion regulation are in line with previous findings, which showed an association between emotion regulation and therapeutic outcome [30]. A recent meta-analysis examined the relationship between emotion regulation and well-being. Across the 35 studies examined, small to moderate correlations were found between various emotion regulation strategies and well-being [20]. The authors discuss that treatments to promote well-being should also focus on promoting emotion regulation.

Regarding potential predictors, it was hypothesized that age, gender, diagnosis, number of previous hospitalisations and living situation are associated with outcome of HT. In addition, work status, civil status, highest degree and variables regarding treatment expectation and evaluation were investigated as potential predictors. Of the investigated potential predictors, three significantly predicted outcome of HT: Former treatment by a psychiatric institution, symptoms of anxiety and hope for improvement.

### Psychiatric History

In line with the hypothesis, it was shown that a history of previous treatments was associated with outcome of HT. Patients without a former treatment by a psychiatric institution had significantly higher well-being and lower symptoms at the time of discharge of HT compared to patients with former psychiatric treatments. The significant covariates of well-being and symptoms at baseline in this regression model indicate that patients who had lower well-being and a higher symptom severity at post-assessment already had a comparatively lower state of health at entry. This finding is consistent with previous results, in which a higher symptom severity was associated with an increased risk of hospital admission during [9, 15, 17] and within 90 days of HT [21]. The results regarding psychiatric history as a relevant predictor for HT outcome are in line with findings of a current systematic review about predictors for HT [6]. Furthermore, according to another systematic review about predictors for readmission to psychiatric inpatient treatment, previous hospitalisation was found to be the most consistently identified predictor [10]. Psychiatric admission within the past 12 month prior to HT may be an indicator for chronicity of a psychiatric disorder and was found to be a predictor for an increased risk for inpatient admission during HT [3, 15].

### Symptoms of Anxiety

Level of Anxiety at baseline was found as a predictor for adaptive emotion regulation at discharge, with higher anxiety at baseline predicting higher adaptive emotion regulation at post-assessment, compared to patients with lower levels of anxiety. The diagnosis of an anxiety disorder has already been associated with the outcome of HT in a previous study. The diagnosis was associated with a lower risk of hospitalization within one year of HT [33]. However, as far as we are aware, this is the first study to identify anxiety as a predictor of outcome in relation to emotion regulation. One possible explanation could lie in the special characteristics of HT. HT makes it possible to include the patient's everyday life under real conditions in the treatment, which also makes it easier, for example, to recognize and deal with factors that trigger crises [16]. The results regarding anxiety as a predictor of emotion regulation can be interpreted on the basis of this particular characteristic of HT. It could be that the treatment succeeded in effectively promoting adaptive emotion regulation strategies in the patient's home environment and everyday life, from which highly anxious patients in particular could have benefited.

### Hope of Improvement

Hope of improvement predicted self-efficacy and adaptive emotion-regulation at post-assessment. Such an effect has also been shown for patients in inpatient treatment, where positive expectations at the beginning of treatment significantly predicted a positive treatment outcome [13]. According to a recent meta-analysis about the effects of patient expectations on improvement in therapy, the association between expectation and effective improvement is partially mediated by the quality of the alliance between patient and therapist [8]. The authors recommend assessing therapy expectation before treatment to be able to use this knowledge to tailor treatment to patient specific needs. This recommendation could be transferred to the assessment of hope within HT.

Contrary to hypothesis, no other predictor was found to be associated with any of the outcome variables. This is not in line with previous findings [6]. For some predictors, other studies also found mixed evidence. For example, living situation was repeatedly investigated as predictor in other studies [7, 15, 19], of which only one found a connection [19]. Regarding age, most previous studies – in line with this study – also could not confirm age as a predictor [7, 15, 17, 22, 25]. However, two studies reported a significant association, with higher age being a risk factor for future admissions to hospital after HT [19, 33]. The authors of both studies discussed that older age could reflect the increasing chronicity of the psychiatric illness.

Another explanation for the lack of significant predictors may be that other variables are more important in HT. For example, a systematic review [6], identified loneliness [22] and family involvement [9] as relevant predictors but both constructs were not assessed in the current study.

One possible reason why outcome could not be predicted by many of the analysed variables could be the indication for HT. It is possible that patients who would not benefit from HT were not included in the treatment in the first place. In a recent study of an HT service in Switzerland, only a small percentage of patients were admitted to an inpatient treatment during HT [32]. According to the authors, this could be interpreted as a consequence of an appropriate indication.

### Strengths and Limitations

The limitations of the study are the following: The sample size was significantly reduced by refusal of participation and dropouts during participation. The reasons for refusing participation were not systematically recorded. In the current study, patients with complete measurements showed a lower symptom burden compared to patients who had to be excluded from the study due to incomplete measurements. This indicates that the mental health status may have influenced the intention to participate in the study, which in turn may have affected the representativeness of the sample and therefore the generalizability of the results. The fact that emotion regulation was not assessed at follow-up represents a deviation from the study protocol and is therefore a clear limitation. The questionnaire was removed from the measurement due to difficulties in data collection at the follow-up time as an attempt to reduce the effort for the measurement and thus reduce the number of missings. The strength of the study is that a wide range of possible predictor variables were analysed. The baseline values of the outcome variables were included in the model as covariates to control for possible confounding effects. Another strength is its methodology. Several different outcome variables were used to assess treatment success, which allowed a more specific analysis of outcome. To our knowledge, the role of emotion-regulation in HT was analysed for the first time.

### Future Research

In future studies, the reasons for refusal to participate should be recorded. This could provide valuable insights into how research with patients during an acute phase of illness can be successful. The current study focused on patient characteristics as predictors. Future research could add variables such as family involvement, loneliness, duration of treatment, medication or emergency call utilisation as potential predictors of treatment success. Future research could address the issue of psychiatric history as a predictor. Which factors lead to the repeated use of psychiatric services and how could treatment services be adapted to the needs of this patient group? In the previous literature, patients who repeatedly seek treatment are also referred to as revolving door patients, whose needs treatment services should be adapted to [29]. In a recent meta-analysis and systematic review about “revolving door” in mental illness, the need for transdiagnostic treatments, targeting overlapping problems (e.g., self-esteem, death anxiety) common to many disorders to prevent relapses was emphasized [23].

## Conclusion

Patients who had previously been treated in a psychiatric institution showed comparatively higher symptom scores and lower well-being at discharge of HT compared to patients without a history of such treatment. A history of previous treatment was discussed as an indicator of the chronicity of psychiatric illnesses. In addition, hope for improvement and symptoms of anxiety were associated with a positive treatment outcome. Apart from these predictors, no patient-related characteristics were found to be associated with the outcome of HT. This may indicate that HT is a suitable and effective form of treatment for a broad spectrum of psychiatric patients.

## Data Availability

Data are available on request from the corresponding author. The data are not publicly available due to privacy or ethical restrictions.

## Appendix

**Table:**

		Adaptive emotion regulation					Maladaptive emotion regulation				
		*B*	*SE*	*β*	*T*	*P*	*B*	*SE*	*β*	*T*	*P*
Sociodemographic variables	Age	0.006	0.010	0.100	0.653	0.517	−0.003	0.010	−0.046	−0.295	0.770
	Sex	0.093	0.263	0.044	0.355	0.724	−0.419	0.266	−0.206	−1.572	0.124
	Civil status *Single/married/divorced/separated*	−0.188	0.300	−0.091	−0.626	0.535	−0.054	0.306	−0.027	−0.177	0.861
	Living situation *Alone/ with others*	0.296	0.295	0.131	1.001	0.323	−0.489	0.297	−0.224	−1.649	0.107
	Work status *Working/ not working*	−0.371	0.281	−0.187	−1.318	0.195	0.363	0.287	0.190	1.266	0.213
	Highest degree	−0.243	0.185	−0.163	−1.310	0.198	0.319	0.189	0.221	1.684	0.100
Psychiatric history	Former psychotherapy *Yes/no*	−0.306	0.264	−0.148	−1.156	0.255	−0.174	0.270	−0.087	−0.646	0.522
	Former treatment by a psychiatric institution *Yes/no*	−0.058	0.245	−0.028	−0.237	0.814	0.526	0.247	0.266	2.127	*0.040*
Well-being	WHO	−	−	−	−	−	−	−	−	−	−
Symptoms	BSCL	−0.168	0.269	−0.119	−0.626	0.535	0.131	0.272	0.096	0.483	0.632
Self-efficacy	SWE	−0.370	0.367	−0.223	−1.010	0.319	0.183	0.277	0.114	0.659	0.513
Emotion regulation	EMOREG *Self-control (adaptive)*	0.604	0.202	0.636	2.992	0.005	0.650	0.186	0.639	3.491	0.001
	*Avoid/distort (maladaptive)*	0.026	0.186	0.025	0.142	0.888	−0.387	0.267	−0.176	−1.447	0.156
Therapy expectation	PATHEV *Hope of improvement*	0.765	0.278	0.336	2.750	*0.009*	−0.099	0.165	−0.087	−0.598	0.554
	*Fear of change*	−0.183	0.169	−0.155	−1.082	0.286	0.103	0.290	0.044	0.356	0.724
	*Suitability*	0.332	0.290	0.137	1.146	0.259	0.650	0.186	0.639	3.491	0.001

Linear Regression Model

*B* Non-standardized coefficient; *SE* Standard error; *β* Standardization factor; *T* t-value; *P* probability value

**Table:**

		Self-efficacy				
		*B*	*SE*	*β*	*T*	*P*
Sociodemographic variables	Age	−0.002	0.006	−0.050	−0.337	0.738
	Sex	0.015	0.154	0.012	0.096	0.924
	Civil status *Single/married/divorced/separated*	0.073	0.176	0.059	0.415	0.681
	Living situation *alone/ with others*	0.176	0.173	0.129	1.017	0.316
	Work status *Working/ not working*	−0.157	0.165	−0.132	−0.948	0.349
	Highest degree	−0.046	0.109	−0.051	−0.421	0.676
Psychiatric history	Former psychotherapy *Yes/no*	−0.001	0.155	−0.001	−0.005	0.996
	Former treatment by a psychiatric institution *Yes/no*	−0.246	0.144	−0.200	−1.713	0.095
Well-being	WHO	−	−	−	−	−
Symptoms	BSCL	−0.003	0.158	−0.004	−0.019	0.985
Self-efficacy	SWE	0.276	0.215	0.276	1.283	0.207
Emotion regulation	EMOREG *Self-control (adaptive)*	0.191	0.118	0.334	1.612	0.115
	*Avoid/distort (maladaptive)*	6.425E-5	0.109	0.000	0.001	1.000
Therapy expectation	PATHEV *Hope of improvement*	0.536	0.163	0.392	3.285	*0.002*
	*Fear of change*	0.024	0.099	0.033	0.239	0.812
	*Suitability*	−0.031	0.170	−0.021	−0.184	0.855

Linear Regression Model

*B* Non-standardized coefficient; *SE* Standard error; *β* Standardization factor; *T* t-value; *P* probability value

**Table:**

	Well-being					Self-efficacy				
BSCL subscales	*B*	*SE*	*β*	*T*	*P*	*B*	*SE*	*β*	*T*	*P*
Hostility	−0.272	0.255	−0.178	−1.067	0.291	−0.121	0.149	−0.156	−0.813	0.421
Anxiety	0.365	0.241	0.302	1.519	0.135	0.094	0.135	0.154	0.698	0.489
Depression	−0.035	0.220	−0.028	−0.161	0.873	−0.235	0.128	−0.359	−1.842	0.072
Paranoid ideation	0.041	0.253	0.029	0.160	0.873	0.071	0.146	0.100	0.488	0.628
Phobic anxiety	0.170	0.168	0.175	1.013	0.316	−0.026	0.099	−0.052	−0.261	0.796
Psychoticism	0.299	0.289	0.215	1.033	0.307	0.086	0.162	0.121	0.529	0.600
Somatization	−0.240	0.224	−0.171	−1.072	0.289	−0.035	0.127	−0.048	−0.274	0.786
Obsessive compulsive	−0.428	0.255	−0.305	−1.678	0.100	0.022	0.145	0.030	0.150	0.882
Interpersonal sensitivity	−0.080	0.241	−0.062	−0.334	0.740	−0.018	0.139	−0.026	−0.126	0.900
Well-being	−0.272	0.255	−0.178	−1.067	0.291	−	−	−	−	−
Self-efficacy	−	−	−	−	−	0.388	0.165	0.388	2.354	0.023

Linear Regression Model

*B* Non-standardized coefficient; *SE* Standard error; *β* Standardization factor; *T* t-value; *P* probability value

**Table:**

	Adaptive emotion regulation					Maladaptive emotion regulation				
BSCL subscales	*B*	*SE*	*β*	*T*	*P*	*B*	*SE*	*β*	*T*	*P*
Hostility	0.051	0.219	0.040	0.234	0.816	−0.013	0.249	−0.010	−0.051	0.959
Anxiety	0.424	0.197	0.415	2.148	0.037	−0.072	0.221	−0.073	−0.327	0.745
Depression	−0.305	0.178	−0.279	−1.711	0.094	0.022	0.190	0.021	0.118	0.906
Paranoid ideation	0.291	0.213	0.246	1.366	0.179	−0.469	0.240	−0.410	−1.956	0.057
Phobic anxiety	−0.213	0.145	−0.260	−1.465	0.150	−0.230	0.162	−0.290	−1.419	0.163
Psychoticism	−0.392	0.238	−0.333	−1.649	0.106	0.177	0.266	0.156	0.664	0.510
Somatization	0.228	0.184	0.192	1.242	0.221	0.095	0.208	0.083	0.457	0.650
Obsessive compulsive	0.010	0.209	0.008	0.048	0.962	0.268	0.234	0.233	1.145	0.258
Interpersonal sensitivity	−0.328	0.202	−0.295	−1.625	0.111	0.435	0.234	0.405	1.861	0.069
Well-being	0.051	0.219	0.040	0.234	0.816	−0.013	0.249	−0.010	−0.051	0.959
Adaptive emotion regulation	0.400	0.125	0.421	3.193	0.003	−	−	−	−	−
Maladaptive emotion regulation	−	−	−	−	−	0.413	0.177	0.407	2.337	0.024

Linear Regression Model

*B* Non-standardized coefficient; *SE* Standard error; *β* Standardization factor; *T* t-value; *P* Probability value

## References

[1] Bandura A. Self-efficacy: toward a unifying theory of behavioural change. Psychol Rev. 1977;84(2):191–215. 847061 10.1037//0033-295x.84.2.191

[2] Barakat A, Blankers M, Cornelis JE, Lommerse NM, Beekman ATF, Dekker JJM. The effects of intensive home treatment on self-efficacy in patients recovering from a psychiatric crisis. Int J Ment Health Syst. 2021;15(1). 10.1186/s13033-020-00426-y. 10.1186/s13033-020-00426-y PMC7789166 33407731

[3] Barbosa JF, Marques JG. The revolving door phenomenon in severe psychiatric disorders: a systematic review. Int J Soc Psychiatry. 2023;69(5):1075–89. 10.1177/00207640221143282. 37209104 10.1177/00207640221143282 PMC10338701

[4] Baumann F, Bergamaschi V, Warnke I, Corbisiero S, Gabriel Felleiter K, Fellmann S, Ludwig F, Riedel A, Znoj H, Schmidt S. Study protocol of an observational study in acute psychiatric home treatment: how does hometreatment work? Identification of common factors and predictors of treatment success. Neuropsychiatrie. 2023;37(4):214–20. 10.1007/s40211-023-00457-0. 36941465 10.1007/s40211-023-00457-0 PMC10703946

[5] Bech P, Olsen RL, Kjoller M, Rasmussen NK. Measuring well-being rather than the absence of distress symptoms: a comparison of the SF-36 Mental Health subscale and the WHO-Five Well-Being Scale. Int J Methods Psychiatr Res. 2003;12(2). 10.1002/mpr.145. 10.1002/mpr.145 PMC6878541 12830302

[6] Bergamaschi V, Baumann F, Warnke I, Corbisiero S, Ludwig F, Riedel A, Gabriel-Felleiter K, Schmidt SJ. Who benefits from acute psychiatric home treatment? A systematic review. Community Ment Health J. 2024;60:1408–1421. 10.1007/s10597-024-01297-0. 10.1007/s10597-024-01297-0 PMC11408559 38940978

[7] Biong S, Ness O, Karlsson B, Borg M, Kim HS. A crisis resolution and home treatment team in Norway: a longitudinal survey study Part 3. Changes in morbidity and clinical problems from admission to discharge. Int J Ment Health Syst. 2012;6(1):17. 10.1186/1752-4458-6-17. 22967433 10.1186/1752-4458-6-17 PMC3459812

[8] Constantino MJ, Coyne AE, Goodwin BJ, Vîslă A, Flückiger C, Muir HJ, Gaines AN. Indirect effect of patient outcome expectation on improvement through alliance quality: a meta-analysis. Psychother Res. 2021;31(6):711–25. 10.1080/10503307.2020.1851058. 33228466 10.1080/10503307.2020.1851058

[9] Córcoles D, Malagón Á, Martín LM, Bulbena A, Pérez V. Home treatment in preventing hospital admission for moderate and severe mentally ill people. Psychiatry Res. 2015;230(2):709–11. 10.1016/j.psychres.2015.08.039. 26343832 10.1016/j.psychres.2015.08.039

[10] Donisi V, Tedeschi F, Wahlbeck K, Haaramo P, Amaddeo F. Pre-discharge factors predicting readmissions of psychiatric patients: a systematic review of the literature. BMC Psychiatry. 2016;16(1):449. 10.1186/s12888-016-1114-0. 27986079 10.1186/s12888-016-1114-0 PMC5162092

[11] Dorman CF, Elith J, Bacher S, Buchmann C, Carl G, Carré G, GarciáMarquéz JR, Gruber B, Lafourcade B, Leitão PJ, Münkemüller T, McClean C, Osborne PE, Reineking B, Schröder B, Skidmore AK, Zurell D, Lautenbach S. Collinearity: a review of methods to deal with it and a simulation study evaluating their performance. Ecography. 2012;36:027–46. 10.1111/j.1600-0587.2012.07348.x.

[12] Franke GH. BSI - Brief symptom inventory von L.R. Derogatis. Göttingen: Hogrefe; 2000.

[13] Grosse Holtforth M, Krieger T, Bochsler K, Mauler B. The prediction of psychotherapy success by outcome expectations in inpatient psychotherapy. Psychother Psychosom. 2011;80(5):321–2. 10.1159/000324171. 21720198 10.1159/000324171

[14] Gühne U, Weinmann S, Arnold K, Atav ES, Becker T, Riedel-Heller S. Akutbehandlung im häuslichen Umfeld: systematische Übersicht und Implementierungsstand in Deutschland. Psychiatr Prax. 2011;28(3):114–22. 10.1055/s-0030-1248598. 10.1055/s-0030-1248598 21207350

[15] Hasselberg N, Grawe R, Johnson S, Saltyte-Benth J, Ruud T. Psychiatric admissions from crisis resolution teams in Norway: a prospective multicentre study. BMC Psychiatry. 2013;13:117. 10.1186/1471-244X-13-117. 23594922 10.1186/1471-244X-13-117 PMC3637541

[16] Hepp U, Stulz N. „Home treatment“ für Menschen mit akuten psychischen Erkrankungen. Nervenarzt. 2017;88:983–8. 10.1007/s00115-017-0355-6. 28573365 10.1007/s00115-017-0355-6

[17] Huang HCH, Taylor M, Carmichael A. The outcomes of home treatment for schizophrenia. BJPsych Bull. 2018;42(6):238–42. 10.1192/bjb.2018.56. 30079860 10.1192/bjb.2018.56 PMC6465209

[18] IBM Corp. Released. IBM SPSS statistics for windows, version 29. Armonk: IBM Corp; 2019.

[19] Kingsford R, Webber M. Social deprivation and the outcomes of crisis resolution and home treatment for people with mental health problems: a historical cohort study. Health Soc Care Community. 2010;18(5):156–464. 10.1111/j.1365-2524.2010.00918.x. 10.1111/j.1365-2524.2010.00918.x 20491965

[20] Kraiss JT, ten Klooster PM, Moskowitz JT, Bohlmeijer ET. The relationship between emotion regulation and well-being in patients with mental disorders: a meta-analysis. Compr Psychiatry. 2020;102:1–14. 10.1016/j.comppsych.2020.152189. 10.1016/j.comppsych.2020.152189 32629064

[21] Leon-Caballero J, Corcoles D, Alba-Pale L, Sabate-Gomez A, Perez E, Monteagudo E, MiguelMartin L, Perez V, Pacchiarotti I. Psychiatric hospitalization at home unit in Spain: clinical and functional outcomes after three years of experience. Actas Esp Psiquiatr. 2020;48(3):138–40. 32905606

[22] Ma R, Wang J, Lloyd-Evans B, Marston L, Johnson S. Trajectories of loneliness and objective social isolation and associations between persistent loneliness and self-reported personal recovery in a cohort of secondary mental health service users in the UK. BMC Psychiatry. 2021;21(1):421. 10.1186/s12888-021-03430-9. 34425767 10.1186/s12888-021-03430-9 PMC8381487

[23] Menzies RE, Richmond B, Sharpe L, Skeggs A, Liu J, Coutts-Bain D. The “revolving door” of mental illness: a meta-analysis and systematic review of current versus lifetime rates of psychological disorders. Br J Clin Psychol. 2014;00:1–19. 10.1111/bjc.12453. 10.1111/bjc.12453 38197576

[24] Murphy SM, Irving CB, Adams CE, Waqar M. Crisis intervention for people with severe mental illnesses. Cochrane Database Syst Rev. 2012;12. 10.1002/14651858.CD001087.pub5. 10.1002/14651858.CD001087.pub5 PMC7052814 26633650

[25] Mötteli S, Schori D, Menekse J, Jäger M, Vetter S. Patients’ experiences and satisfaction with home treatment for acute mental illness: a mixed-methods retrospective study. J Ment Health (Abingdon, England). 2020;1–8. 10.1080/09638237.2020.1803233. 10.1080/09638237.2020.1803233 32772614

[26] Mötteli S, Schori D, Schmidt H, Seifritz E, Jäger M. Utilization and effectiveness of home treatment for people with acute severe mental illness: a propensity-score matching analysis of 19 months of observation. Front Psychiatry. 2018;9. 10.3389/fpsyt.2018.00495. 10.3389/fpsyt.2018.00495 PMC6191514 30364109

[27] Schulte D. Messung der Therapieerwartung und Therapieevaluation von Patienten (PATHEV). Z Klin Psychol Psychother. 2005;24(3):176–87. 10.1026/1616-3443.34.3.176.

[28] Schwarzer R, Jerusalem M. Generalized self-efficacy scale. In: Weinman J, Wright S, Johnston M, editors. Measures in health psychology: a user’s portfolio. Causal and control beliefs. Windsor: NFER-NELSON; 1995. p. 35–7.

[29] Sentissi O, Bartolomei J, Moeglin C, Baeriswyl-Cottin R, Rey-Bellet P. The Geneva model of crisis intervention. A retrospective study. J Psychol Psychother. 2014;4(4). 10.4172/2161-0487.1000150.

[30] Sloan E, Hall K, Moulding R, Bryce S, Mildred H, Staiger PK. Emotion regulation as a transdiagnostic treatment construct across anxiety, depression, substance, eating and borderline personality disorders: a systematic review. Clin Psychol Rev. 2017;57:141–63. 10.1016/j.cpr.2017.09.002. 28941927 10.1016/j.cpr.2017.09.002

[31] Stulz N, Wyder L, Maeck L, Hilpert M, Lerzer H, Zander E, Kawohl W, Holtforth MG, Schnyder U, Hepp U. Home treatment for acute mental healthcare: randomised controlled trial. Br J Psychiatry. 2020;216(6):323–30. 10.1192/bjp.2019.31s. 30864532 10.1192/bjp.2019.31

[32] Stulz N, Wyder L, Grosse Holtforth M, Hepp U. Is hometreatment for everyone? Characteristics of patients receiving intensive mental health care at home. Community Ment Health J. 2021;58:231–9. 10.1007/s10597-021-00814-9. 33735397 10.1007/s10597-021-00814-9

[33] Werbeloff N, Chang C-K, Broadbent M, Hayes JF, Stewart R, Osborn DPJ. Admission to acute mental health services after contact with crisis resolution and home treatment teams: an investigation in two large mental health-care providers. Lancet Psychiatry. 2017;4(1):49–56. 10.1016/S2215-0366(16)30416-3. 27979719 10.1016/S2215-0366(16)30416-3

[34] Znoj H. Entwicklung des Fragebogens zur Emotionsregulation in schwierigen Lebenssituationen (EMOREG). Unpublished manuscript, Universität Bern; 2000.

